# A Working-Class View of Our Hospitals

**Published:** 1888-01-21

**Authors:** A. W. Webster

**Affiliations:** Chairman of Workmen's Governors, Sunderland Infirmary


### A Working-Class View of Our Hospitals.

By A. W. Webster, Chairman of Workmen's Governors, Sunderland Infirmary. (*Continued from p.* 264.)

Merely suggesting a systematic contribution from workmen is likely to be of little value without hints as to how so desirable an end can be reached. Perhaps the best method to follow in my treatment of this is to quote a few questions recently put to me by hospital managers and others, and then to answer them as clearly as possible. Of course it must always be understood that local influences and circumstances differ so much that no suggestions of mine are likely to meet the requirements of every case, yet plain answers to plain questions may supply hints that practical-minded hospital managers will be able to add to or take from, and adapt to their particular needs.

I. "What method of procedure would you recommend to successfully inaugurate your scheme in places where it had never been tried?"

The constitution or rules of your hospital must be altered so as to allow workmen to sit on your committee. (The basis of representation and principle of election will be found further on in this article.) You must enlist the sympathy and help of public men, and lose no opportunity of teaching workmen that hospitals specially exist for their class, and ought to be as systematically supported as their trades unions or friendly societies. The aid of the local press is exceedingly valuable. After the scheme has had due publicity arrangements might be made for a meeting of the workmen employed in any particular factory, to be held either in the latter part of the dinner-hour, or immediately after work ceases at night. A notice calling this meeting should be posted in the works a few days before, to allow the workmen to talk the matter over in the interval. Such meetings should always be held within the works, a shed or workshop being quite suitable, and the active co-operation of the employer or manager will do much to ensure success. Moreover, the best-known and most popular members of the local Hospital Committee or other active patrons of local charities should be chosen to bring the matter before the workmen.

If these plans be judiciously carried out and not too hastily acted on, I think the workmen will generally be ready to pass a resolution at the meeting referred to, agreeing to contribute their penny per week to the local charity or charities.

II. "How are the pennies collected? If more than one system is used, which do you find most effectual?"

Employers of labour render valued aid in the collection, and in most of our large works the penny is simply deducted from the men's wages (at their own request), and afterwards handed to the infirmary secretary. Indeed, the infirmary penny is so thoroughly established, that many of our ship-builders, engineers, and others have a column ruled in their wages-books to allow of its regular deduction, and to be able to account satisfactorily for all money passing through their hands in this manner. In some places one of the men stands near the pay-table and collects the coppers from his fellow-workmen after they receive their wages. This man is usually the workmen's infirmary governor for that factory or workshop. Other systems of collecting are either rare or inefficient, and call for no comments.

III. "What remuneration or privilege is given to collectors?"

Cashiers and other officials working under the direction of their employers, neither ask nor expect remuneration. No doubt the work of the wages clerk is slightly increased by keeping off the infirmary penny, but in most cases it is simply a labour of love, and the employers or officials who refuse to contribute their mite of labour to so laudable an object as this are rare indeed. The same applies to the workmen who collect the pennies at the pay-tables. The only return they seem to care for is a fair share in the management of the institution.

IV. "Are the workmen represented on the Hospital Committee? If so, how are they elected, and how is their interest in the institution maintained?"

Workmen have nominally assisted in the management of Sunderland Infirmary for many years, but previous to its being a free hospital they were simply elected by the usual well-to-do infirmary subscribers, and had no real connexion with the working classes. It has now become a pretty generally accepted principle that representation should accompany taxation, and as soon as our workmen commenced to contribute regularly to hospital support, it was found necessary to carry this into practice, and a basis of representation was fixed.

The first set of resolutions passed by the Annual Court of Governors bearing on this matter is as follows:—

"That the workmen in each friendly society, shipyard, factory, or workshop, subscribing in the aggregate more than £10 yearly to the funds of the infirmary shall be entitled to nominate one of their number as a governor of the institution for the year following such payment."

"That four members of the General Committee may be nominated by the working men."

"That four representatives of the working men (beside those on the General Committee) be elected members of the Special Committee for the election of the honorary medical officers."

The standard sum of £10 is regularly reached by the work-people employed in about sixty factories, &c., in and near the town. These sixty places appoint each a representative who is styled a workmen's governor of the infirmary. These representatives meet quarterly (and much more frequently when special schemes are on hand), and are constituted the Court of Workmen's Governors. Their powers are limited by the above resolutions, but they appoint their own chairman and officers, and are regulated by a proper code of rules. The workmen's representatives on the General Committee and those on the Special Committee for the election of the honorary medical officers, are elected from this body, thus maintaining the true principle of representation throughout.

It will readily be seen that this Court of Workmen's Governors is a thoroughly representative body, with easy means of reaching and influencing the whole of the working classes, and although the regular source of income is now so well established, they find it always possible to legislate on ways and means for increasing it. Special schemes are initiated by them and carried out much on the same line as those organised by the workpeople's agencies in other towns. For instance, a Workmen's Industrial Exhibition was successfully carried through by them in the spring of the present year, £400 being handed to the Executive Committee of the infirmary as the result of this workmen's effort alone.

A new wing is at present being added to the infirmary, and some of the more influential ladies of the town intend holding a large bazaar next year to help to pay for its cost. Here again the workmen have stepped in, and have readily agreed to supply a stall of goods equal in value to some hundred pounds, whilst the yearly bazaars and other special efforts on behalf of our children's wards are, to some extent, directly and indirectly, supported by our workmen. Whether the Hospital Sunday collection be gathered in church, chapel, or Sunday school, it is almost sure to find a special advocate in some of the past or present workmen's representatives, and the Friendly and Trades Societies' demonstration in aid of the hospital funds is now a well-established yearly event. Dramatic and concert parties, football and cricket clubs, constantly find members of their associations clamouring for charity performances. The influence of the workmen's representative is everywhere felt, and with so many spheres of activity there is seldom much difficulty in maintaining the interest of the working classes.

V. "To what extent are other charities in the town supported by the workmen?"

The infirmary being the chief institution in the eyes of the workmen, it receives the largest support. Other institutions, however, are partially maintained by them, the following plans being used in different places. Some have a special subscription now and again for a particular charity, this being in addition to the regular infirmary penny, whilst others contribute more than a penny per week, and give the extra subscription to the smaller charities. Others again subscribe the penny only, and some give a proportionate subscription to the amount of wages earned, that is, those having under ten shillings a week contribute a halfpenny; those above ten shillings and under thirty give one penny; and those above thirty shillings give twopence. The workmen then decide yearly what proportion of their subscriptions shall be given to each institution.

Sunderland has as yet experienced little difficulty in centralising its charitable institutions, and this division of the workmen's subscriptions in limited in its operation, and principally refers to a small Accident Home and Dispensary on the north side of the river, to which the workmen have contributed sums varying from £250 to £450 per annum.

(*To be continued.*)

